# Surface-Imprinted Polymer Coupled with Diffraction Gratings for Low-Cost, Label-Free and Differential *E. coli* Detection

**DOI:** 10.3390/bios16010060

**Published:** 2026-01-13

**Authors:** Dua Özsoylu, Elke Börmann-El-Kholy, Rabia N. Kaya, Patrick Wagner, Michael J. Schöning

**Affiliations:** 1 Institute of Nano- and Biotechnologies (INB), Aachen University of Applied Sciences, Campus Jülich, 52428 Jülich, Germany; oezsoylu@fh-aachen.de (D.Ö.); boermann-el-kholy@fh-aachen.de (E.B.-E.-K.);; 2 Laboratory for Soft Matter and Biophysics, Department of Physics and Astronomy, KU Leuven, B-3001 Leuven, Belgium; patrickhermann.wagner@kuleuven.be; 3 Institute of Biological Information Processing (IBI-3), Research Centre Jülich GmbH, 52425 Jülich, Germany

**Keywords:** surface-imprinted polymer, *E. coli* detection, simulation, dual-functionality, diffraction gratings

## Abstract

Surface-imprinted polymer (SIP)-based biomimetic sensors are promising for direct whole-bacteria detection; however, the commonly used fabrication approach (micro-contact imprinting) often suffers from limited imprint density, heterogeneous template distribution, and poor reproducibility. Here, we introduce a photolithography-defined master stamp featuring *E. coli* mimics, enabling high-density, well-oriented cavity arrays (3 × 10^7^ imprints/cm^2^). Crucially, the cavity arrangement is engineered such that the SIP layer functions simultaneously as the bioreceptor and as a diffraction grating, enabling label-free optical quantification by reflectance changes without additional transduction layers. Finite-difference time-domain (FDTD) simulations are used to model and visualize the optical response upon bacterial binding. Proof-of-concept experiments using a differential two-well configuration confirm concentration-dependent detection of *E. coli* in PBS, demonstrating a sensitive, low-cost, and scalable sensing concept that can be readily extended to other bacterial targets by redesigning the photolithographic master.

## 1. Introduction

In the current global landscape, characterized by high mobility and interconnectedness, the rapid spread of diseases caused by pathogenic bacteria poses a significant threat to contemporary healthcare, as well as environmental and food safety [1,2]. The World Health Organization (WHO) estimates that annually 600 million people fall ill and 420,000 die from consuming contaminated food, with most cases linked to pathogenic bacteria [2,3]. Furthermore, the WHO revised its list of bacterial priority pathogens in 2024, emphasizing that microorganisms represent the highest threat to global health [4]. According to the updated list, *Enterobacterales*, including several antibiotic-resistant *E. coli* strains, were classified in the “critical group” (the highest priority), which are frequently resistant to multiple drugs and challenging to manage, resulting in unfavorable outcomes in severe infections like sepsis. Addressing this global challenge demands new-generation diagnostic tools to detect, identify, and track the pathogenic bacteria effectively.

Conventional methods for bacterial pathogen identification, such as biochemical staining and culture-based techniques (e.g., colony-counting methods), still remain the gold standard [5]. Nevertheless, these methods are time-consuming (usually require 2–4 days for identification), laborious (need trained personnel), and have limited applicability, as only certain species can be successfully cultured [5,6,7,8,9]. Alternative methods, such as enzyme-linked immunosorbent assay (ELISA) and polymerase chain reaction (PCR) are also widely used for detecting pathogenic bacteria; however, these techniques are usually expensive, time-consuming due to the sample enrichment/preparation procedures, and often unsuitable for field testing since they necessitate specialized instruments, skilled staff, or centralized laboratory facilities [6,8,10,11,12]. Therefore, emerging sensing techniques that allow for point-of-care detection of whole bacteria in the field are becoming increasingly popular. Biosensors that utilize specific biorecognition elements, including antibodies, aptamers, or surface/molecularly imprinted polymers (SIPs or MIPs), represent a promising strategy for the detection of whole bacteria [6,9,12,13,14]. SIP-/MIP-based biomimetic sensors have garnered considerable interest due to their durability, cost-efficiency, and extended shelf life [9,12,14,15,16,17,18,19,20].

The process of molecular imprinting typically involves the copolymerization of functional monomers alongside the targeted component using a suitable cross-linker. Hence, the target component (such as bacteria) is used as a template. Once polymerization is complete, the template is removed, resulting in the formation of specific binding cavities (recognition sites) that are complementary in shape and size to the original template. Applying whole bacteria for imprinting is the highly preferred method for generating cavities (as artificial receptors) that are specific to target bacteria. The most direct and effective means of producing these bacterial imprints is to perform micro-contact imprinting [21,22,23]. In this method, firstly, a stamp is prepared by attachment of the template bacteria to a substrate material (e.g., glass or polydimethylsiloxane (PDMS)). Afterwards, stamping is carried out by pressing this stamp into a pre-polymerized polymer matrix (such as photopolymers or polyurethane), usually spin-coated on a preferred planar sensor surface, e.g., interdigitated electrodes. This procedure leads to the formation of sensor chips based on SIPs [24]. The resulting bacterial cavities (imprints) match the size/shape of the target bacteria (physical recognition) and also retain imprints of bacterial surface components, like lipopolysaccharides, which are important for chemical recognition. The synergistic combination of both chemical and physical recognition enhances affinity towards the target bacteria, thereby improving detection capabilities [23,25].

While SIP-based sensors fabricated by micro-contact imprinting (using template bacteria) demonstrated significant potential for direct detection of whole bacteria, they still face limitations: the heterogeneous distribution of bacteria on the stamp surface—uncontrolled spatial arrangement and areal density—can result in irreproducible cavity formation on the sensor surface, causing significant inter-experimental variability [24,26]. This inconsistency needs the establishment of standards for scalable production. Additionally, the method relies on labor-intensive bacterial cultivation for template preparation, also requiring specialized facilities and skilled personnel, even though only certain species can be successfully cultured [5]. Handling critical pathogenic species further demands stringent biosafety protocols to mitigate infection risks [25]. Moreover, mechanically induced cellular flattening during imprinting also distorts cavity geometry, potentially reducing the capture of the target bacteria due to geometric mismatch [27]. In addition, achieving a high areal cavity density (e.g., >6.5 × 10^6^ cavities per cm^2^) on the sensor surface, which is important for high sensor sensitivity, is difficult due to bacterial cell aggregation on the stamp surface [18,24]. To address these challenges, we recently reported an initial step toward “template bacteria-free” fabrication of SIP-based biomimetic sensors [28]. This method employs a PDMS-based positive master stamp, containing photolithographically defined bacterial mimics to generate structural analogues of conventional bacteria-imprinted cavities. This approach enabled the fabrication of SIP layers on quartz crystal microbalance (QCM) chips and interdigitated electrodes (IDEs), with the possibility of adjusting the areal cavity density, all without relying on real bacterial templates. While this fabrication method provides a promising foundation for SIP-based sensors targeting whole-bacteria recognition, realizing its full potential requires further innovations—such as tailoring cavity density and orientation to introduce additional functional capabilities into the SIP layer and developing cost-effective alternatives to existing systems using QCM chips, IDEs, or surface plasmon resonance (SPR) devices [19,29].

In this work, we could obtain 3 × 10^7^ biomimetic *E. coli* cavities per cm^2^ by employing a “template bacteria-free” fabrication method based on photolithographic mimics of template bacteria. Moreover, the distribution and orientation of cavities were arranged in a way that they can serve as diffraction gratings for optically detecting captured bacteria. Hence, this combination allows the SIP layer to perform a dual function—carrying cavities for binding bacteria and reading out this binding event by means of a reflectance change as an optical sensor signal. Furthermore, simulation studies using the finite-difference time-domain (FDTD) method were carried out to optimize the sensor’s optical performance as well as to create a useful model for visualization of the *E. coli* detection event. Additionally, binding of *E. coli* inside the cavities was studied by atomic force microscopy (AFM) and scanning electron microscopy (SEM). The feasibility of *E. coli* detection was demonstrated with differential measurements against PBS solution (without *E. coli*) thanks to spatially arranged double measurement wells on the same sensor chip. The developed biomimetic sensor chip for *E. coli* detection ensures low-cost, label-free, and quantitative measurements.

## 2. Materials and Methods

### 2.1. Fabrication of the Master Mold

To achieve biomimetic structural analogues of *E. coli* imprints, a master mold was designed by CAD (computer-aided design) software (KLayout) and fabricated by means of photolithographic patterning of a photoresist on a substrate (glass wafer). For designing this master mold, the geometry of the patterns (cavities) was defined according to the physical characteristics of the rod-shaped *E. coli* cells, as reported in the literature [30]. In addition, our recent work [28] revealed that a longer length (around 2.7 µm) of the cavities results in higher *E. coli* capturing efficiency compared to shorter length (1.6 µm and 2.1 µm) cavities. Therefore, the width and the length of the cavities were adjusted to 1 µm and 2.7 µm, respectively. To maximize the areal density of the cavities and to achieve high diffraction efficiency, the patterns were closely located to each other to form grating periods with 3 × 10^7^ cavities per cm^2^. The total size of the pattern area was defined as 45 mm × 45 mm so that it could fit the size of a 3-inch wafer (76.2 mm diameter).

**Figure 1:**
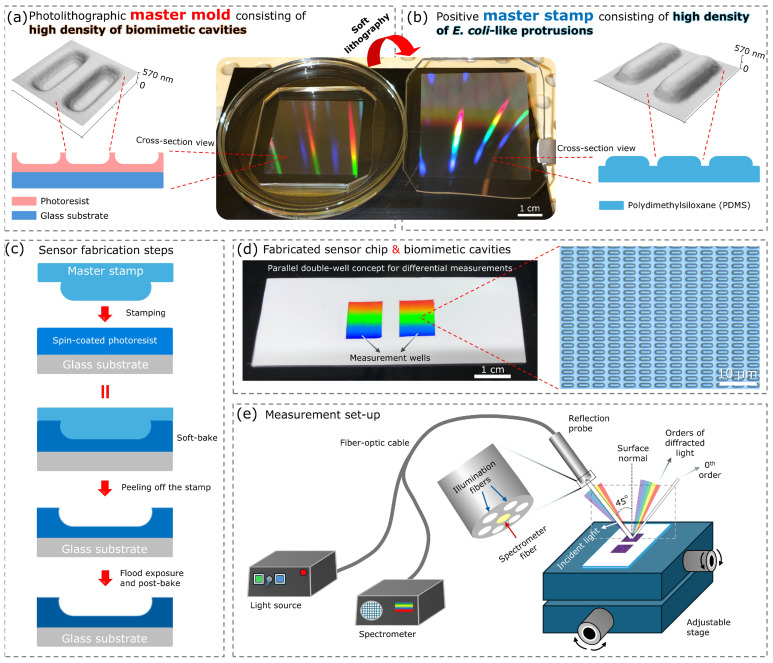
(**a**) Master mold consisting of high density of biomimetic *E. coli* cavities fabricated by direct laser writing lithography. (**b**) PDMS-based positive master stamp with high density of *E. coli*-like protrusions fabricated by pattern transfer by molding (soft-lithography from the master mold). (**c**) Sensor fabrication steps based on surface imprinting using the master stamp. (**d**) Fabricated sensor chip setup based on biomimetic *E. coli* imprints. A double-well was defined on the sensor chip thanks to a hydrophobic tape (white layer) having two open windows (1 cm × 1 cm). The digital microscopy image on the right side shows the cavities. Both wells consist of the same type of SIP layer, allowing differential measurements. (**e**) Measurement set-up based on reflective diffraction gratings.

Direct laser writing lithography was employed to fabricate the master mold using a photoresist (AZ 5214E [JP], diluted 1:0.476 using methoxy-propyl acetate, Microchemicals GmbH, Ulm, Germany) in the positive mode. Firstly, the photoresist was first spin-coated (4000 rpm, 30 s) on a glass wafer (3 inch, 0.5 mm thick, Borofloat 33, Siegert Wafer GmbH, Aachen, Germany). Thereafter, a soft bake at 105 °C for 1.5 min was carried out. Using a direct laser writer device (PicoMaster 150, Raith GmbH, Dortmund, Germany), the patterns were written with a 405 nm laser source of the device. For this, an exposure energy of 40 mJ/cm^2^ (step resolution: 275 nm, spot size: 550 nm) was applied to achieve geometrical similarity with *E. coli* only on the surface of the photoresist without involving the flat glass wafer underneath. After writing, the development was performed with tetramethyl-ammonium hydroxide (TMAH, 2.38% in H_2_O, AZ 726 MIF, Microchemicals GmbH, Ulm, Germany) for 1 min without reversal-bake (as positive mode was applied). The obtained patterns (master mold) underwent a flood exposure at 200 mJ/cm^2^, followed by a post-bake (3 min) at 120 °C to enhance the adhesion and chemical stability. Figure 1a demonstrates the final form of the master mold inside a glass Petri dish (100 mm). The patterned area (a rectangle of 45 mm × 45 mm) with a rainbow effect can be recognized at the middle of the mold.

### 2.2. Fabrication of the Master Stamp

As shown in Figure 1a,b, the master stamp was obtained from the master mold using soft lithography. For this, the pattern transferring was performed by molding using a two-component polydimethylsiloxane (PDMS) elastomer (Sylgard 184, Dow, Midland, MI, USA). Hence, PDMS was prepared at a standard mixing ratio (10:1) according to the instructions of the manufacturer, and the mixture was then vacuum-degassed for 40 min in a desiccator. Thereafter, the degassed mixture (without bubbles) was poured on the fabricated mold located in a glass Petri dish (100 mm). To prevent any possible air bubbles that can be trapped in the cavities, degassing with a vacuum was repeated. Using a hot plate, the curing of the PDMS elastomer was performed at 100 °C for 45 min. As shown in Figure 1b, after the curing, the PDMS elastomer was cut to the desired size (larger than the size of the patterned area) with a razor blade, and the positive master stamp was obtained after separating (peeling off) it from the mold. For imprinting, this large positive master stamp was further cut into smaller pieces depending on the desired imprinting size and geometry. The remaining PDMS elastomer inside the Petri dish was not removed to provide a pool for the next molding application (hence, minimizing the required amount of PDMS) as well as to protect the mold underneath.

### 2.3. Surface Imprinting and Sensor Chip Fabrication

Figure 1c describes the basic steps of the sensor chip fabrication based on surface imprinting. A photopolymer resin (AZ 5214E [JP], Microchemicals GmbH, Ulm, Germany), fabricated master stamp, and glass microscopy slide (75 mm × 25 mm) were used for chip fabrication. Firstly, the photopolymer resin (based on cresol novolak resin and diazonaphthoquinone sulfonic acid ester) was diluted with propylene-glycol-mono-methylether-acetate with a dilution ratio of 1:0.476. This dilution ensured an approximately 600 nm thick layer after it was spin-coated (4000 rpm for 30 s) on the glass microscopy slide, which was cleaned with acetone and isopropanol in an ultrasonic bath for 10 min. Stamping was performed right after the spin-coating process by pressing the master stamp onto the photopolymer resin-coated glass surface. A soft bake was then applied at 105 °C for 2 min. After the structures cooled down to room temperature (20 °C), the stamp was peeled off to obtain the patterns (imprinted cavities). The obtained patterns underwent a post-processing by a flood exposure of 300 mJ/cm^2^ and post-bake at 120 °C for 3 min. This procedure is important to enhance the chemical stability and adhesion of the SIP layer.

To fabricate non-imprinted polymer (NIP) samples consisting of a flat and non-patterned photopolymer resist surface, the same procedure was followed; however, a non-patterned (without *E. coli*-like protrusions) PDMS sample was used as a stamp instead of using the developed master stamp. A hydrophobic tape (Microfluidic Diagnostic Tape, 9965, 3M, St. Paul, MN, USA) was used to passivate the area on the sensor chip surface that is not used for measurements. For this, two rectangular holes with the size of 1 cm by 1 cm were opened in the middle of the tape, and the tape was then attached onto the areas to be passivated. In this way, a double-well with SIP surface was formed. Depending on the application mentioned in the next sections, two different sensor chips were fabricated: i. the chip consists of two wells with only the SIP layer (for signal measurements), and ii. the chip consists of two wells, with one of them being the SIP layer while the other is the NIP layer (for analysis of cell capturing). As an example, Figure 1d shows the resulting sensor chip (with only SIP layers) together with a zoomed-in digital microscopy image revealing the imprinted cavities on the wells. Furthermore, the ability of the wells to hold the analyte solution thanks to the hydrophobic barrier is also shown in Figure S1 (Supplementary Information).

Note on terminology: While the developed sensor is based on a surface imprinting approach using a master stamp with photolithographically defined bacterial shapes, it does not yet include chemical imprinting elements such as molecular recognition sites, and it is not a molecularly imprinted polymer. Therefore, the produced polymer layer is more accurately described as a geometrically surface-imprinted polymer (SIP), and the non-imprinted polymer (NIP) control corresponds to a smooth, flat surface without any cavity features.

### 2.4. Characterizations of the Chip Surface

For quality control and general characterization of the pattern transfer and imprinting processes, a digital microscope (VHX-7000, Keyence, Itasca, IL, USA) was used. In addition, scanning electron microscopy (SEM) was employed to explore the patterns in more detail. Before SEM imaging, the samples were coated with a 5 nm thick platinum–palladium layer. Thereafter, an SEM device (JSM-7800F, Jeol GmbH, Freising, Germany) was utilized to record SEM images. The topography of the patterned surfaces was explored by atomic force microscopy (AFM). Hence, with a silicon-based cantilever (ARROW-NCR-20, NanoWorld AG, Neuchâtel, Switzerland) attached to an AFM device (BioMat Workstation, JPK Instruments, Berlin, Germany), the AFM imaging was performed under non-contact mode. The free and open-source software Gwyddion [31] was used for visualization and analysis of AFM data.

### 2.5. Bacteria Cultivation and Preparation

*Escherichia coli* K12 (DSM 498, DSMZ GmbH, Braunschweig, Germany) was used as a model organism to analyze the performance of the developed sensor chip. *E. coli* K12 was cultivated at 37 °C and 150 rpm (KS 4000i control, IKA-Werke GmbH, Staufen im Breisgau, Germany) in lysogenic broth (LB) medium [32]. In order to apply the bacterial cells as targets for the developed sensor chips, they were first incubated in 10 mL LB medium in 100 mL flasks with baffles for 16–24 h. Then, 1 mL of this pre-culture was used to inoculate 50 mL of LB medium in 500 mL flasks with baffles. After reaching the stationary phase, the optical density was determined at 600 nm (Ultrospec 2100 pro, GE Healthcare UK Limited Amersham Place, Little Chalfont, Buckinghamshire, UK) and the total cell number of the cultivated batch was determined by means of the Thoma counting chamber (0.02 mm chamber depth). The cells were harvested by centrifugation (10 min, 6000× *g*, Megafuge 1.0 R, Heraeus Instruments, Hanau, Germany), the supernatant was discarded, cells were washed three times in sterile phosphate-buffered saline (PBS), pH 7.4, and finally re-suspended in 10 mL PBS buffer so that they could be applied on the sensor chips. Subsequently, the desired cell concentrations between 1 × 10^3^ to 1 × 10^9^ cells/mL were obtained by preparation of suitable dilutions and used in the following experiments.

### 2.6. Analysis of Cell Capturing

The *E. coli* capturing performance of the developed sensor chip was evaluated by a differential analysis using the parallel double-well measurement concept on the same sensor chip. Unlike the sensor chip demonstrated in Figure 1d, where both wells consist of an SIP layer, this time, one of the two wells was prepared as an NIP surface. This configuration allows comparison of the cell capture ability of SIP and NIP surfaces to calculate the “imprinting factor” (IF, the ratio between the number of bacteria bound on the SIP layer and bacteria bound on the NIP layer).

By adding 0.3 mL of *E. coli* K12 cell solution (5 × 10^8^ cells/mL in PBS) to each well of the sensor chip, the SIP and NIP surfaces were exposed to the bacteria (at room temperature, around 20 °C) for 15 min. Thereafter, the wells were rinsed with PBS solution. To fixate the cells for SEM and AFM imaging, a fixation solution (2.5% glutaraldehyde in PBS) was added to the wells and incubated at room temperature overnight. The wells were then rinsed with ultrapure water to exchange the glutaraldehyde solution and dried using nitrogen gas. Thereafter, the microscopic analysis using SEM and AFM was performed. To calculate and compare the number of *E. coli* cells captured by the surfaces, the cells that stayed on the surfaces were analyzed by SEM images and recorded from different spots on the surfaces (87 images for each NIP and SIP surface). For both SIP and NIP surfaces, the SEM recordings were carried out with the same imaging parameters (e.g., magnification); the *E. coli* cells in each obtained image were counted by using the software ImageJ (version 1.54g) [33]. To evaluate topography-driven (geometry-based) selectivity of the SIP surface, we performed the same capture/SEM analysis using *Saccharomyces cerevisiae* and *Priestia megaterium* as non-target microorganisms. These species were selected because they present a pronounced size/shape mismatch relative to the *E. coli* K12-mimic cavities (*S. cerevisiae*: ovoid and 5–10 µm in diameter; *P. megaterium*: often in pairs/short chains and length of up to 4 µm and a diameter of 1.5 µm), allowing us to isolate the contribution of geometric matching to capture.

### 2.7. FDTD Simulations

To evaluate the sensor’s optical characteristics and response while capturing *E. coli* cells, as well as to form a useful model for this detection event, we have carried out the finite-difference time-domain (FDTD) method, which is a 3-D full-wave electromagnetic solver frequently used in nanophotonics to efficiently simulate light interacting with a variety of materials and optical devices [34]. In FDTD, the simulation domain is the area defined by the simulation boundaries and segmented into a mesh grid. During the execution of an FDTD simulation, the electromagnetic (EM) fields are determined based on Maxwell’s equations within each mesh cell, with the computations being stepped through time repeatedly. Spatial discretization enables the modeling of intricate geometries and structures, while temporal discretization tracks the progression of EM fields as time unfolds.

For FDTD simulations, we used the commercial software (Lumerical, ANSYS, Inc., Canonsburg, PA, USA). To build a more realistic simulation scenario, the detailed 3-D structure of the SIP layer obtained from AFM imaging was imported into the simulation environment to utilize it as a surface structure. For this, first, AFM imaging data (high-resolution scanning with the size of 8 µm to 8 µm) of the SIP layer was converted to a text file (as a matrix) by means of Gwyddion software (version 2.61) (Czech Metrology Institute, Brno, Czech Republic) [31]. Then, by applying the import function (as a surface) of the Lumerical software, this text file was used to create the SIP surface for the simulations. Refractive index data of the photopolymer resin (as a SIP material) was added as an n,k material (n, refractive index and k, extinction coefficient) to the material database; thereafter, it was defined as a material for the SIP layer. The substrate material was selected as glass from the material database of Lumerical. From the software’s object library, a “rounded-cylinder” was used to define an *E. coli* cell with a rod-shaped structure. The bacteria were specified as a dielectric material with a refractive index value of 1.388 [35,36]. The width and length of *E. coli* were adjusted to 1 µm and 2.5 µm, respectively, as average values reported in the literature [30,35].

### 2.8. Measurement Set-Up

Figure 1e depicts the optical measurement set-up for the detection of *E. coli* cells. In this set-up, thanks to the fiber-optic cables of the reflection probe, the polychromatic (multiple wavelength) light source incident on the sensor surface consists of periodic patterns (imprinted cavities) that work as diffraction gratings. The diffraction gratings split the incident light into multiple beam paths, which causes light of different wavelengths (component wavelengths) to propagate in different directions. The reflected light is collected by a reflection probe (with spectrometer fiber) at the same angle as the illumination fiber and is transferred to the spectrometer. The spectrometer analyzes the intensity of the reflected light as a function of wavelength (intensity spectra).

As a light source, we utilized a deuterium–halogen lamp (wavelength range: 210–2500 nm, DH-2000, Ocean Optics, Inc., Orlando, FL, USA). A fiber-optic cable with 400 µm fiber core size (wavelength range: 200-1100 nm, QR400-7-SR-BX, Ocean Optics, Inc., Orlando, FL, USA) was used as a reflection probe. The probe’s fiber bundle consists of six illumination fibers (they connect to the light source) around one “read fiber” (the spectrometer fiber, the single-fiber leg connects to the spectrometer) (Figure 1e, middle image). A spectrometer (wavelength range: 535–700 nm, EQPro, Ocean Optics, Inc., Orlando, FL, USA) was used to record reflected light spectra (intensity of the reflected light as a function of wavelength). With a holder, the position of the reflection probe was adjusted in a way that the angle of incident light (the angle between the incident ray and the normal) was kept constant at 45°. In addition, using an adjustable movement stage (*x*,*y*,*z*), where the sensor chip was fixed on it, the distance between the chip surface and the reflection probe was adjusted to 2 mm. The stage was also operated for horizontally moving the sensor chip in a way that the probe could sequentially illuminate both wells on the sensor chip. Hence, this optic configuration allows differential measurements.

For *E. coli* detection, the sensor chip (see Figure 1d) with two identical wells (both consisting of the SIP layer) was first fixed on the stage, and then “reference measurements” (initial signal without any incubation on the two identical wells) were recorded. Thereafter, one of the two wells (“*E. coli* measurement well”) was incubated with 0.3 mL of *E. coli* solution (10^3^ cells/mL in PBS) for 15 min, while the same amount of PBS solution was applied for the other well (“control well”) for 15 min for differential measurements. After the incubation, the wells were rinsed (one time) with deionized (DI) water to prevent possible salt crystals that could remain on the sensor surface. Signal measurements were recorded from both wells after the wells were dried with nitrogen gas. Thereafter, the same periods of incubation, rinsing, drying, and measurement steps were applied to the “*E. coli* measurement well” for each *E. coli* solution with higher concentrations (ranging from 10^4^ cells/mL to 10^9^ cells/mL in PBS), while the same procedure was applied consecutively to the other well (“control well”) but using only PBS solutions (instead of *E. coli* cells) each time to reveal possible effects of PBS solution, drift, or fluctuations in the sensor signal.

The signal recordings were operated by the software (OceanView, version 2.0.8, Ocean Optics, Inc., Orlando, FL, USA) of the spectrometer. Integration time as a key data acquisition parameter was adjusted to five seconds. Just after each recording, the shutter of the light source was closed (the light source was not switched off) until the next recording. During the measurements, the background spectrum was also recorded before each measurement while the shutter was closed, and it was subtracted from the measurement signal to eliminate possible involvement of the environmental background light. In this way, “relative intensity” versus “wavelength” curves were achieved for each measurement. To better observe the signal shift over the application of *E. coli* cells, each curve was subtracted from the curve of the “reference measurement”. For differential measurements, calculation details are provided in Section S1 (Supplementary Information). The limit of detection (LOD) was calculated based on the signal-to-noise criterion. The standard deviation of the blank signal ($\sigma_{\mathrm{blank}}$) was determined from measurements performed in the absence of bacterial cells. The sensitivity (*b*) was obtained from the slope of the linear calibration curve relating the sensor response to the logarithm of the bacterial concentration. The LOD was then calculated according to [37] as:

(1) $$\mathrm{LOD}=\frac{3\sigma_{\mathrm{blank}}}{\left|b\right|}$$

where $\sigma_{\mathrm{blank}}$ represents the standard deviation of the blank signal, and *b* is the calibration slope (sensitivity) expressed in counts/dec; thus, the equation yields the detection limit in the logarithmic domain, which was converted to concentration units (cells/mL) using the fitted calibration function.

## 3. Results and Discussion

### 3.1. Characterizations of Surface Topographies

Molding with soft lithography using a master mold (Figure 1a) provides unique advantages to obtain numerous replicas—positive master stamps—(Figure 1b,c) without the need for photolithography again. Figure 1d shows the resulting sensor chip (with only SIP layers) together with a zoomed-in digital microscopy image revealing the imprinted cavities on the wells. Figure 1e depicts the optical measurement set-up for the detection of *E. coli* cells.

**Figure 2:**
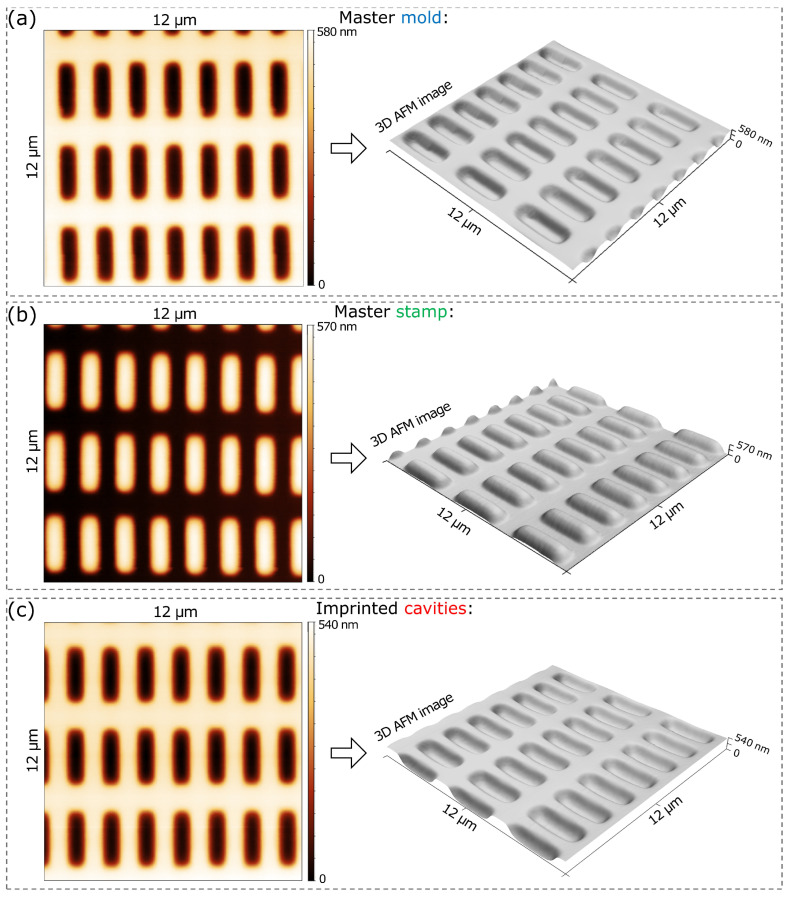
The 2-D and 3-D AFM images showing surface topography and geometry of fabricated (**a**) master mold, (**b**) master stamp, and (**c**) imprinted cavities of the SIP layer.

AFM imaging was employed to investigate detailed characteristics of the acquired functional layers, including the depth, distribution, and three-dimensional structure of the patterns. Figure 2a and Figure 2b show 2-D and 3-D AFM images of the fabricated master mold and master stamp, showing the surface topography and geometry of the cavities and *E. coli*-like protrusions, respectively. The images indicate a successful pattern transfer from the mold to the stamp characterized by high topography similarity. Similar to the cavities on the mold surface, the height, width, and length of the protrusions on the stamp surface were 570 nm, 1080 nm, and 2700 nm, respectively. These biomimetic protrusions of *E. coli* cells on the master stamp exhibit a high degree of geometric resemblance to the actual *E. coli* bacteria, as reported in [30].

The digital microscopy image in Figure 1d and AFM images in Figure 2c confirm the successful replication of an *E. coli* SIP layer consisting of imprinted cavities. These images reveal a uniform and flawless transfer of the photolithographic patterns to the sensor surface. The structures (cavities) were intact and well-ordered. Most importantly, a curved geometry, necessary to replicate rod-shaped *E. coli* cells, was successfully achieved. Only a slight decrease (around 40 nm) in the depth of the cavities was observed after imprinting. On the other hand, other topographical characteristics were well preserved. As a result, the depth, width, and length of the cavities on the sensor’s SIP surface were also around 540 nm, 1080 nm, and 2700 nm, respectively. These achieved cavity geometries on the SIP sensor surface provide high structural similarity with that of the imprints using real *E. coli*, as reported in the literature [18,27,38].

One of the primary objectives of producing SIP-based sensors is to create cavities with the highest possible areal density. However, in the conventional micro-contact imprinting method, the challenge of achieving higher imprint densities with a uniform distribution stems from the overlapping/clustering of bacteria cells, which occurs due to their aggregation when applied to the stamp surface at higher concentrations. Therefore, such formations can result in a suboptimal signal response [24]. By employing the “Dickert’s imprinting” technique (which is a well-established method for SIP fabrication), our team previously managed to achieve an imprint density of 6.5 × 10^6^ imprints per cm^2^ after conducting multiple optimization studies using *E. coli* bacteria [18]. In our previous report [28], we were able to develop a SIP surface with 10^7^ imprints per cm^2^ using a similar template cell-free imprinting method based on a photolithographic master stamp. Notably, in the current study, we finally reached 3 × 10^7^ imprints per cm^2^ in a standard and reproducible manner without employing any template bacteria.

### 3.2. Evaluation of *E. coli* Capturing Ability

**Figure 3:**
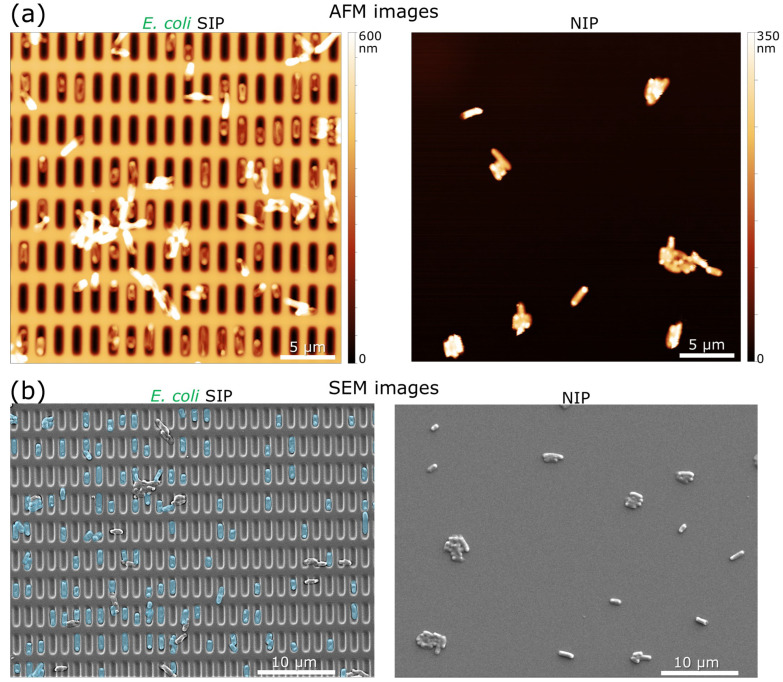
Representative 2-D AFM height images (**a**) and SEM images (**b**) show the captured *E. coli* K12 cells on the SIP (left) and NIP (non-imprinted) (right) surfaces. Note: the imaging was performed after 15 min exposure with *E. coli* cells (5 × 10^8^ cells/mL in PBS). The surfaces were washed with PBS solution before fixing the remaining cells for microscopic examinations.

It is clear that a sensor surface based on a SIP with a high bacteria-capturing ability compared to one made of a non-imprinted polymer (NIP) should be reached. Figure 3a displays representative 2-D AFM topography images of the SIP and NIP surface after they were exposed to *E. coli* cells (5 × 10^8^ cells/mL in PBS) for 15 min while Figure 3b shows the corresponding SEM images. To enhance the visibility of *E. coli* captured in the cavities of the SIP layer, false-coloring was applied in the SEM image (Figure 3b(left)). Contrary to the cells captured on the SIP surface, the images show that most cells on the NIP surface clustered instead of spreading more uniformly on the surface.

From the 2-D AFM image (Figure 3a(left)) and SEM image (Figure 3b(left)), it can be seen that the *E. coli* cells are predominantly located within the cavities. The variation in cell capturing between SIP- and NIP-based surfaces was also studied using SEM imaging at the border regions (where imprinted and non-imprinted surfaces meet), as shown in Figure S2 (Supplementary Information).

**Figure 4:**
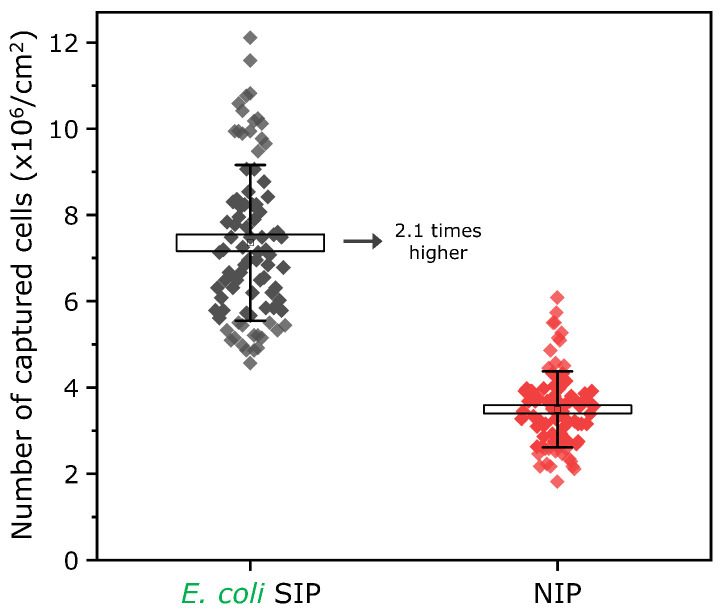
Evaluation of *E. coli*-capturing efficiency on the sensor surface with biomimetic *E. coli*-imprinted cavities and comparison with an NIP (non-imprinted) layer. The figure shows the number of captured *E. coli* K12 cells on the surfaces (SIP, left and NIP, right) after 15 min exposure with cells (5 × 10^8^ cells/mL in PBS).

A statistical assessment of the *E. coli*-capturing effectiveness of the SIP layer in comparison to the NIP layer was conducted for a total of 174 SEM images (after *E. coli* cells were incubated on the surfaces for 15 min). Each image was examined by tallying the total number of cells captured by the surfaces (SIP and NIP), and the “imprinting factor” (the ratio of *E. coli* cells adhered to the SIP layer compared to those adhered to the NIP layer) was determined. These findings are presented in Figure 4. Upon comparing the SIP and NIP after a 15 min incubation time with the bacteria, the imprinting factor is approximately 2.1.

In addition, since some *E. coli* bacteria adhered to the edges of the cavities, the proportion of bacteria located directly within the cavities was also quantified. The analysis revealed that approximately 81% of the bacteria detected on the SIP surface were located within the imprinted cavities. Moreover, the cavity occupancy ratio—defined as the number of bacteria-occupied cavities per 100 cavities on the SIP surface—was found to be 34%. These results further demonstrate the significant contribution of the imprinted cavities to bacterial binding on the SIP surface. In addition, regarding the obtained imprinting factor (IF = 2.1) and cavity occupancy (34%), we note that whole-cell imprinting factors reported for *E. coli* vary widely and are strongly dependent on the adopted definition and readout (signal-based versus capture-based), as well as assay parameters, such as incubation duration, concentration range, and washing protocol; therefore, direct numerical comparison across platforms is inherently limited. Nevertheless, IF values on the order of ∼2–3 are frequently observed for bacteria-sized targets even in chemically assisted imprinting strategies [38,39,40]. In contrast, the cavity occupancy ratio is not a commonly reported metric in the SIP literature, where percentage-type values are more often given as imprint/template surface coverage during fabrication rather than post-binding cavity filling [41,42]. In our system, however, the number, geometry, and periodicity of the *E. coli*-mimic cavities are well-defined, enabling a straightforward quantification of post-incubation occupancy. Accordingly, the observed 34% occupancy also indicates that a substantial fraction of the high-density cavity array remains accessible and active for capture under the short-incubation assay conditions used here.

Numerous studies have reported strategies to enhance cell occupancy efficiency in microwells, often targeting single-cell trapping. These improvements were achieved through various approaches, including optimizing microwell geometry—such as width, size, and depth—for *E. coli* [43,44] and Jurkat cells [45]; using meniscus-aided docking [46] and incorporating microfluidics for yeast cells [46] and K562 cells [47]; applying dielectrophoresis to capture *E. coli* [48] and cancer cells [49]; using dual-nested microwell arrays with AI (artificial intelligence) analysis for HCT116 cells [50]; employing soft-lithographic surfaces for Ramos cells [51]; adjusting injection flow rates for HeLa cells [52]; and refining microwell diameter and seeding protocols for RBL-1 and 3T3 fibroblasts [53]. All of these studies mostly used relatively large microwells and emphasized general trapping efficiency rather than geometric shape matching. Only a few attempts have demonstrated the advantage of bacteria shape–size matching cavities. For example, Helbig et al. [54] showed that microwells only slightly larger than *E. coli* improved bacterial occupancy. In the previous work [28], shape–matching cavities of varying lengths (1600 nm, 2100 nm, and 2600 nm) were compared, and it was observed that longer cavities captured more *E. coli*. Based on these findings, a 2700 nm cavity length was preferred in this study. Nonetheless, further optimization tailored to different bacterial species is necessary, as microbial traits such as motility, surface chemistry, and outer membrane composition can significantly affect capture behavior [44].

To assess the selectivity of the SIP-based gratings, we quantified the microorganism-capturing efficiency (number of captured cells per cm^2^) for morphologically distinct species, namely *E. coli* K12, *Saccharomyces cerevisiae* (yeast), and *Priestia megaterium* (Gram positive). The SIP surface captured approximately $\left(7.35\pm 1.80\right)\times 10^{6}$*E. coli* cells/cm^2^, while the numbers of captured *S. cerevisiae* and *P. megaterium* cells were much lower, at $\left(2.3\pm 2.5\right)\times 10^{5}$ and $\left(4.0\pm 5.7\right)\times 10^{5}$ cells/cm^2^, respectively. SEM images (Figure S3, Supplementary Information) corroborate this pronounced selectivity, showing only sparse attachment of yeast and *P. megaterium*, which predominantly locate themselves onto (yeast) or partially within (*P. megaterium*) individual cavities. Since *S. cerevisiae* cells are typically 3–4 µm in diameter (i.e., larger than the cavity dimensions), and *P. megaterium* cells are usually longer than the cavities and often occur in pairs or short chains, there is a clear geometrical mismatch between these cells and the imprinted topology. We therefore attribute the significantly reduced capture of *S. cerevisiae* and *P. megaterium* (compared with *E. coli*) to this geometry mismatch, which contributes to the overall selectivity of the developed SIP surface.

One of the main conclusions of these findings is that the quantity of captured *E. coli* bacteria is significantly greater in the biomimetic cavities (SIP surface), which possess distinct geometric characteristics similar to rod-shaped *E. coli* cells, in comparison to NIP surfaces. When the geometrical pattern features of the surface, such as the shape and dimensions of the biomimetic imprints (cavities), are comparable to the size of the bacterial cells, this enhances their attachment within those patterns [28], which is also demonstrated by the experiments with *S. cerevisiae* and *P. megaterium*. Such cavities could cause better adhesion as they maximize contact area while helping bacteria effectively protect themselves within the cavities. In this context, as structural analogues of real *E. coli* imprints, the obtained biomimetic cavities may stimulate such behavior, which reflects the contribution of geometry–dependent physical recognition in SIP-based *E. coli* sensing. The results obtained could serve as a foundation for future research focused not only on whole bacteria sensors utilizing molecular surface-imprinted polymers but also on other technologies aimed at the direct identification of target bacteria. For instance, the engineered biomimetic cavities might be combined with various biorecognition components (such as antibodies and aptamers) to further improve sensing efficacy.

### 3.3. Simulations

**Figure 5:**
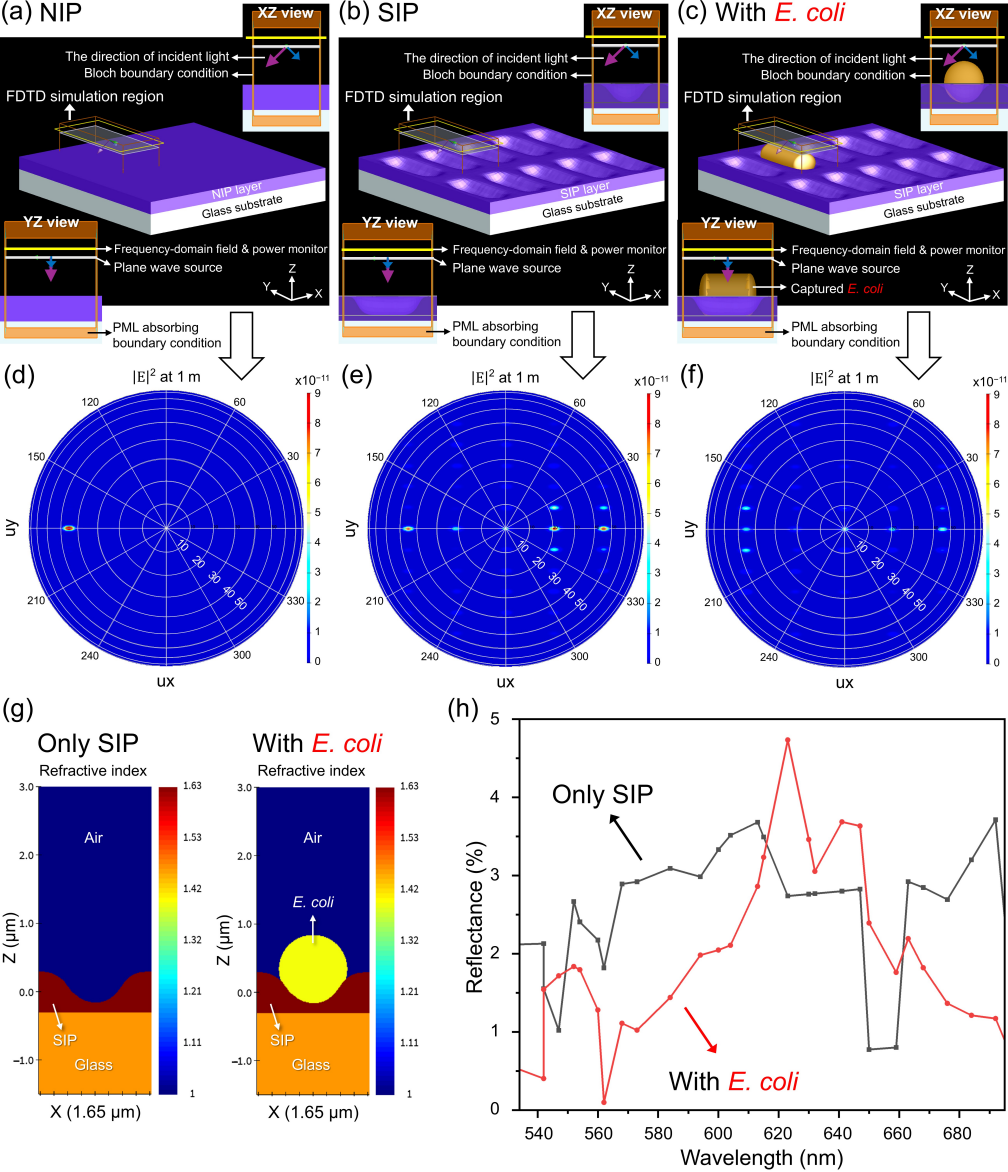
FDTD simulation to characterize and visualize the *E. coli* detection event using an SIP-based sensor chip. (**a**–**c**) Schematic diagrams of FDTD simulation setups for different surface properties: NIP (non-imprinted polymer), SIP (surface-imprinted polymer) and SIP with *E. coli* cells, respectively. PML: perfectly matched layer. (**d**–**f**) Far-field profiles showing the electric field intensity (proportional to |E|^2^) of the reflected light projected to a hemisphere with a radius of 1 m from the corresponding sensor chips: NIP, SIP, and SIP with *E. coli* cells. (**g**) Demonstration of 2-D cross-sectional refractive index distributions of 2-D simulation regions. (**h**) Comparison of reflectance changes as a function of wavelength over admission of an individual *E. coli* cell.

To understand the *E. coli* detection principle of the developed sensor chip and, furthermore, to create a useful model to visualize this detection event, we have carried out finite-difference time-domain (FDTD) simulations. Figure 5a–c shows the schematic diagram of FDTD simulation setups for different surface properties: NIP (non-imprinted polymer), SIP (surface-imprinted polymer), and SIP with *E. coli* cells, respectively.

The SIP layer consists of periodically imprinted cavities. The most effective method for calculating the response of the entire chip that exhibits such periodic structures is to include a single periodic unit cell within the simulation area. Therefore, as can be seen in Figure 5a–c, the FDTD simulation region was located in a way that it only includes one of these unit cells. Hence, the width (towards *x* direction) and length (towards *y* direction) of the simulation region were 1.65 µm and 3.85 µm, respectively. The cross-sectional images (*xz* view and *yz* view) in Figure 5a–c show, in addition to the structures, two important components of the setup (light source and field and power monitor) as well as boundary conditions. The type of light source (0.9 µm above the SIP or NIP surface) was defined as a plane wave, as it is frequently used in diffraction gratings. As an input to the simulation program, the incident light propagates along the *z*-axis. The angle of incident light (angle theta) was set to 45° (see the purple arrow from the plane wave, Figure 5a–c), perpendicular to the longitudinal direction of the cavities (see also Figure 1d,e). The polarization angle was defined as 0° (p-polarization, see the blue arrow). As it is relevant to measure the reflected (rather than the incident or transmitted) field and power, a “frequency-domain field and power monitor” was placed behind the plane wave source (1.1 µm above the SIP or NIP surface). The monitor was used to measure the electric field components ($E_{x}$, $E_{y}$ and $E_{z}$) and the transmitted power (T) of the reflected light. Here, “E” represents the electric field (its unit is V/m) data as a function of position and wavelength, while “T” indicates the total amount of power (its unit is W) transmitted through the monitor, normalized to the source power. As an example, by considering the total injected source power (e.g., 1 W), a T value of 0.04 means that 4% of the optical power injected by the source passed through the monitor after it is reflected from the surface. This percentage (4%) is named the total reflectance. As the propagation of the plane wave source is at an angle (45°), the Bloch boundary condition (instead of the periodic boundary condition) was used in the *x* and *y* directions (see Section S2.1 in the Supplementary Information for more detailed information). In the z direction, the perfectly matched layer (PML) and adsorbing boundary condition with a “steep angle” profile were employed to absorb incident light without significant reflections. The mesh refinement was generated by using an “auto non-uniform” meshing algorithm. Considering simulation time, this setting was selected to minimize the effects of numerical dispersion because of the uneven surface topography of the cavity region. As the SIP/NIP structure is defined as an n,k material and as the plane wave source angle is at 45° (a non-normal incident angle), each simulation was performed at a single source wavelength.

Figure 5d–f shows simulation results as far-field profiles indicating the electric field intensity (|E|^2^) of the reflected light (with a wavelength of 584 nm) projected to a hemisphere with a radius of 1 m from the corresponding sensor chips: NIP, SIP, and SIP with *E. coli* cells. The far-field projections visualize the electric field as a function of the variables $U_{x}$ and $U_{y}$ (electric field unit vectors), which are the *x* and *y* components of the unit direction vector. As the easiest way to interpret the simulation, it can be imagined that the sensor chip/simulation area is located in the middle of the hemisphere, and far-field data of the reflection was plotted as if we look straight down on the hemisphere. In this way, multi-parametric/complex far-field data of the reflection (diffraction angles, modes and their intensities) can easily be interpreted. For instance, as it is an NIP surface (no cavities, not a diffraction grating), there is only one visible diffraction order in Figure 5d, and it is located on the $U_{y}$ line (0 azimuthal angle) and between the 40th and 50th longitudinal rings, meaning the angle of 45° to the *z*-axis. This indicates the 0th diffraction order reflected from the surface at 45° to the *z* axis just as if the grating were a plane mirror. The total reflectance of the NIP layer was calculated as 0.67%, and it was accumulated on the 0th diffraction order. On the other hand, in Figure 5e, other diffraction orders are also visible where a sensor chip with an SIP layer (instead of a NIP layer) was simulated. For example, from left to right on the $U_{y}$ line, the angles of the 0th, 1st, 2nd, 3rd, and 4th diffraction orders are approximately 45°, 21°, 0°, 21° and 45°, respectively. In addition to these orders on the $U_{y}$ direction (0 azimuthal angle), other orders characterized with different azimuthal angles also become visible. The total reflectance of the SIP layer was calculated as 2.59%, and this reflected power was accumulated mostly on 0th, 3rd, and 4th diffraction orders. At the same time, as described in Figure 1e, for the real measurements, the reflection probe is located at 45° to the surface normal, and its spectrometer fiber is also located at the same place (with an angle of 45°). Therefore, only the light reflected back at the same angle as its incident light can be measured. Due to this, in the above-mentioned configuration, where the 584 nm light source is injected at 45° to the surface with a 1650 nm grating spacing (length of each period), only the 4th diffraction order (with 45°) can be measured. Figure 5f shows the simulation of the diffraction orders after the binding of an *E. coli* cell inside the SIP cavity. Compared to Figure 5e (only SIP layer), the presence of the cell decreases the intensities of almost all orders. In addition, the total reflectance decreased to 1.94%, and this reflected power was rather equally distributed to all diffraction orders. Furthermore, Figure 5g visualizes refractive index distributions of a 2-D cross-section (*xz* plane towards the middle of the cavity) at 584 nm.

Reflectance changes as a function of wavelength were also simulated to compare the findings with real measurements. The details of this simulation work are given in Section S2.2 in the Supplementary Information. Figure 5h shows the comparison of reflectance changes over the capturing of an additional *E. coli* cell. Accordingly, the spectra in Figure 5h represent the simulated reflectance for the experimental collection geometry (45° return direction), rather than the hemispherically integrated total reflectance. The figure (Figure 5h) indicates that the reflectance mostly decreases at the wavelength range from 560 nm to 610 nm and from 660 nm to 695 nm. These findings indicate that the local refractive index change due to the *E. coli* cell binding (Figure 5g), which causes a shift in reflectance towards lower values, especially at the above-mentioned specific wavelength regions (Figure 5h).

### 3.4. *E. coli* Measurements

**Figure 6:**
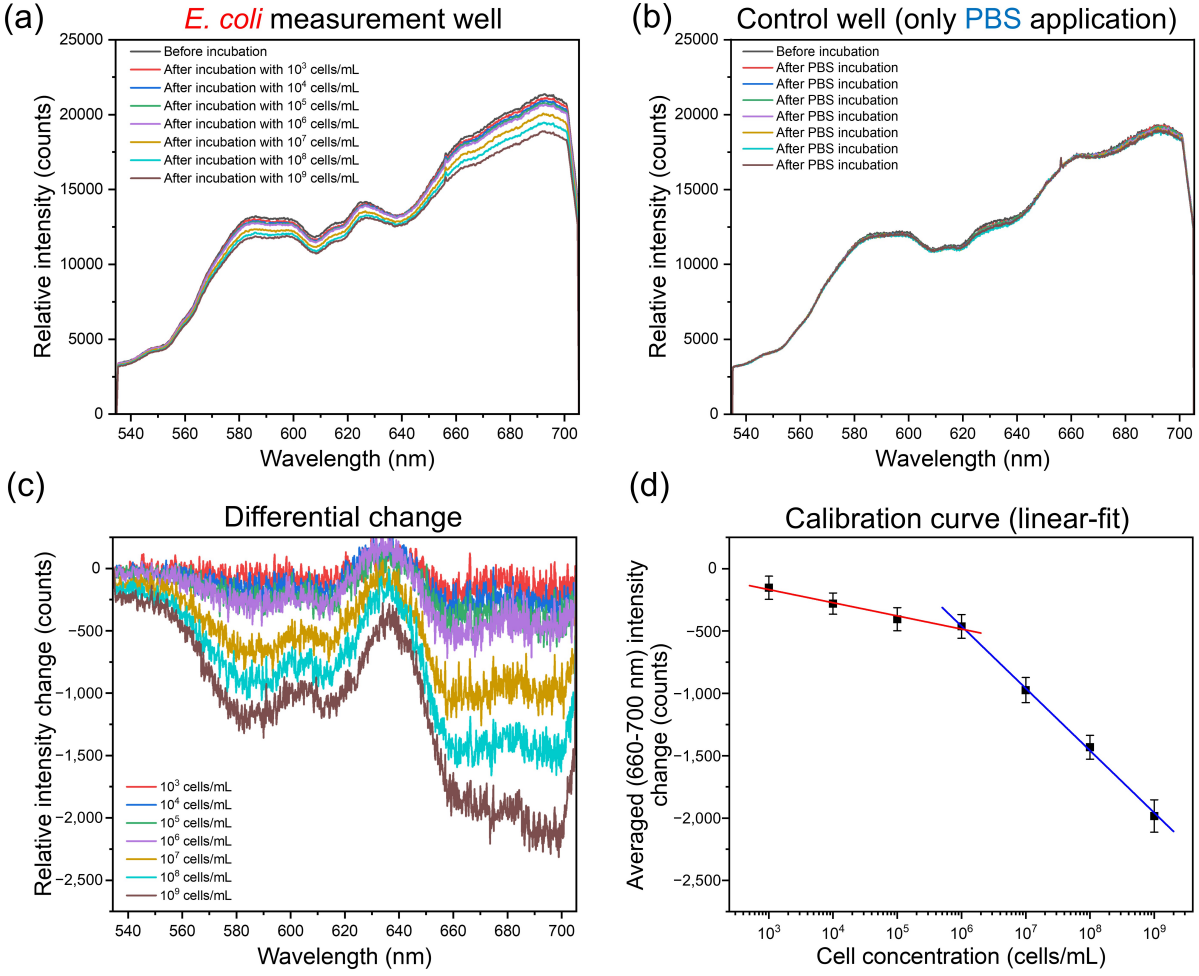
Differential signal measurement. One of the double-wells is used for *E. coli* cell detection, while the other is applied for control measurements without cells but only PBS solution. (**a**) Relative intensity spectra over the applied *E. coli* cells at different concentrations (from 10^3^ to 10^9^ cells/mL in PBS). (**b**) Relative intensity spectra over the PBS applications. (**c**) Relative intensity change due to the cell applications, obtained by subtracting the initial signal curve (before incubation) from other curves in (**a**). Note: The corresponding signal changes in the control well (see (**b**)) were also subtracted to differentiate the sensor signal. For calculation details of the differential change, see Equation (S1) in Section S1 of the Supplementary Information. (**d**) Calibration curve corresponding to the applied cell concentrations from 10^3^ to 10^9^ cells/mL in PBS.

As shown before, the SIP-based receptor layer with a high density of imprinted cavities on the sensor surface is capable of binding *E. coli* cells. Figure 1d and the attached video (see Supplementary Information) show visible reflected colors (rainbow) due to the diffraction of broadband light on the receptor layer of the sensor chip consisting of imprinted cavities. For the quantitative detection of *E. coli* cells (10^3^ to 10^9^ cells/mL in PBS), the two wells (see Figure 1d) were utilized. Here, one well was loaded with cells, whereas the other well (control well) was used for “control measurements”, where only PBS solutions were applied to reveal possible drift or fluctuations in the sensor signal. The measurement results are overviewed in Figure 6. For the corresponding calculation of the differential signal, see Equation (S1) in Section S1 of the Supplementary Information.

The curves in Figure 6a,b show the relative intensity spectra of the reflected light from the sensor surface over the applied *E. coli* cells and over the admission of PBS solution (control well), respectively. The relative intensity measured from the SIP layer (before incubation, Figure 6a) at, e.g., 584 nm, was 278 times higher than that of the NIP surface (Figure S4, Supplementary Information). This is in good agreement with the simulation findings, where the SIP layer shows higher total reflectance and higher electric field intensity in its diffraction orders than that of the NIP layer (see Figure 5d,e). In Figure 6a,b, several peaks were observed in the intensity spectra. In general, similarly to the simulation results (Figure 5h, SIP curve), the intensity signals were low in the wavelength range of around 534–560 nm, while a higher increase in intensity values was seen in the wavelength ranges of around 560–584 nm and 660–692 nm. In Figure 6a, the curves were shifted towards lower intensity values, with increasing *E. coli* cell concentrations ranging from 10^3^ to 10^9^ cells/mL. In the control well, where only the PBS solution was applied each time, the signal values remained fairly stable (Figure 6b).

To better visualize the signal change, the initial signal curve (before incubation) was subtracted from the relative intensity curves obtained after *E. coli* application. In addition, the corresponding signal changes in the control well were also taken into account to calculate the differential signal (see Equation (S1) in Section S1 of the Supplementary Information). Hence, Figure 6c shows the “relative intensity change” versus “wavelength” curves as a differential measurement. Two distinctive valley regions (drop at relative intensity) in the curves were observed at the wavelength ranges of around 560–620 nm and 660–700 nm. These findings are in good agreement with the simulation results (Figure 5h), where a distinct separation between the curves of “only SIP” and “with *E. coli*” was observed within these wavelength ranges. Even if the simulated model demonstrates decent similarity with the real measurements, in the simulation the effects of possible cell/fabrication imperfections, the surface roughness of the model *E. coli* cell and its multiple heterogeneous structures with different refractive indexes were not studied in detail. This simplification in our simulation could result in variations between the experiment and the simulation.

Figure 6d shows the calibration curves (relative intensity change versus applied cell concentration) obtained by linearly fitting the intensity changes. The linear relationship (calibration curve) between *E. coli* concentration and response signal (intensity change) was found as two sections with different slopes corresponding to a low and high concentration range. In classical non-covalent MIPs, the heterogeneous distribution of binding sites—caused by incomplete monomer-template complex formation—typically results in both low- and high-affinity binding sites, which might result in such two-section behavior [55,56,57]. While this effect is well studied for small molecules, no systematic studies exist for larger templates such as whole bacteria, which present a complex variety of surface molecules, antigens, and receptors. In our current approach, only the geometric features of the bacteria were transferred to the biomimetic sensor using a PDMS stamp that was not functionalized with bacterial surface epitopes. The observed change in slope across the concentration range may therefore result from the proportion of *E. coli* in the cavities altering depending on *E. coli* incubation concentration: at lower concentrations, fewer *E. coli* cells may reach and settle into the imprint cavities, while at higher concentrations, the likelihood of cells entering and occupying these cavities increases, leading to a stronger and more consistent signal. Similar behavior with *E. coli* cells was also previously reported [9]. From the calibration curves (Figure 6d), detection sensitivities were calculated to be 105 ± 12 counts/dec and 498 ± 13 counts/dec for *E. coli* concentrations in the range of 10^3^ to 10^6^ cells/mL and 10^6^ to 10^9^ cells/mL, respectively. These findings of the differential measurement indicate that the sensor chip responds to the presence of the target cells (*E. coli* K12 as a model organism) in the analyte solution. The results also demonstrate a broader measurable concentration range compared to a previously reported SIP-based QCM-D sensor, which was limited to a detection range of 10^7^ to 10^9^ *E. coli* cells/mL [28]. On the other hand, signal noise remains a limiting factor, particularly in the low concentration range (Figure 6c) and should be carefully considered when interpreting sensor performance. Using differential signal measurements and the 3σ criterion, the limit of detection (LOD) of the sensor was estimated to be $6.4\times 10^{3}$ cells/mL.

Placing the sensitivity and limit of detection (LOD) of the present sensor into context, it should be noted that reported *E. coli* detection limits vary widely across biosensing technologies and are strongly influenced by assay format (label-free versus labeled/amplified), capture chemistry (antibody/aptamer versus synthetic receptors), and sample preparation or preconcentration steps. Representative antibody- and aptamer-based platforms can reach very low limits of detection (often down to single-digit CFU/mL) but typically rely on biological recognition elements and/or signal amplification, for example, an antibody-conjugated electrochemical sensor reported an LOD of 1 CFU/mL [58], and a digital SERS aptamer sensor reported an LOD of 0.657 CFU/mL with mixed-bacteria interference tolerance [59]. Hybrid double-recognition schemes combining aptamers with imprinted polymers have also been demonstrated, e.g., an Apta-MIP electrochemical sensor reported an LOD of 2 CFU/mL for *E. coli* [9]. In comparison, label-free optical whole-cell sensors without enrichment more commonly operate in the ∼$10^{3}$ CFU/mL regime; as a representative example, a long-period fiber-grating biosensor reported reliable *E. coli* detection down to $10^{3}$ CFU/mL [60]. Within this landscape, the present work emphasizes a scalable and durable SIP-based concept in which the cavity array simultaneously serves as the bioreceptor and the diffraction grating for optical readout, avoiding biological capture reagents and labels while offering a practical route toward further performance gains through assay-flow optimization and/or optional preconcentration.

Briefly, the introduction of *E. coli* to the SIP-based biomimetic sensor chip surface results in their capture onto this specific design structure (Figure 3 and Figure 4), which induces a change in the local refractive index within the cavities of the SIP surface (bacteria have a lower refractive index than that of the SIP material). This causes a predictable shift in the reflectance toward lower values, and, correspondingly, a decrease in the intensity signal of the reflected light (Figure 6a,c,d). Such polymeric diffraction grating-assisted detection principles based on local refractive index changes upon binding targets have already shown great potential for different sensing applications [61,62,63,64,65]. For instance, recently, a PDMS-based diffraction grating surface (as grooves) was functionalized with a molecularly imprinted polymer (MIP) layer to recognize C-reactive protein [65]. For signal recording, almost the same setup with a 45° angle of incident light and receiving probe was used. Upon binding of the target protein, a decrease in the intensity of reflected light due to the change in refractive index was reported. In addition to similar reports [62,65], some other works used the transmission principle for measurements, where the transmitted power is recorded after the light passes through the gratings [61,64].

“Template bacteria”-based SIP fabrication methods such as the “Dickert’s imprinting” approach [29,66,67] are well-established to obtain biomimetic sensors for bacteria detection. Such biomimetic sensors usually consist of additional functional elements such as quartz crystal microbalance or surface plasmon resonance (SPR) sensor chips [19,29]. In this study, the distribution/orientation of the cavities could be adjusted uniformly and periodically, which allows them to serve as diffraction gratings for optically detecting captured bacteria. This would hardly be possible using conventional “template bacteria”-based micro-contact imprinting techniques. Furthermore, contrary to plasmonic sensors using metallic gratings, such as grating-coupled SPR sensors [68], the reported sensor chip does not require deposition of any additional metal layer, and the SIP layer is formed on a simple glass microscopy slide as a substrate; hence, providing a low-cost alternative. The large-scale production of surface-imprinted polymer-based bacteria detection sensors remains a significant, yet unmet, target in the field. Our fabrication strategy employs direct laser writing photolithography only once to generate a functional master mold. This mold is used to produce numerous positive replicas via simple soft lithography that eliminates the need for repeated “rather costly” photolithography steps. Unlike conventional bacteria imprinting techniques, it also allows precise control over the areal density, depth, distribution, and orientation of bacterial imprints without the use of live bacteria. This could provide a scalable pathway toward mass production.

While selectivity experiments were not the primary focus of this study, the designed sensing mechanism inherently supports a degree of physical selectivity. The topographical match between the *E. coli*-shaped cavities and target cells enhances the contact area and promotes preferential adhesion, while excluding bacteria of different geometries. Our experiments with the non-target microorganisms *S. cerevisiae* and *P. megaterium* (see Section 3.2) demonstrate that topography-driven effects are a key contributor to the selectivity of our system. Consistent with this, Stilman et al. [38] also reported that spherical and smaller *S. aureus* bacteria exhibited minimal adhesion to *E. coli*-shaped cavities compared to rod-shaped bacteria. In addition, Helbig et al. [54] demonstrated that *S. epidermidis*, being smaller and spherical, showed poor adherence to cavities fabricated for *E. coli*. Beyond geometry, differences in the intrinsic refractive indices of bacterial types may further enhance discrimination in our optical readout. Gram-negative bacteria generally exhibit higher bulk refractive indices than Gram-positive species due to differences in cell wall composition [69]. Even among rod-shaped, Gram-negative bacteria, selective detection might be possible due to intrinsic refractive index differences, which could translate into distinct measurable intensity shifts, generating optical signatures that provide an additional selectivity dimension [69,70]. However, the present study aimed primarily to demonstrate a new sensing concept that integrates bacteria-shaped cavity arrays into a diffraction grating, enabling real-time optical detection based on reflectance changes. Future work will focus on extending this concept toward chemical selectivity by functionalizing the stamp surface with outer membrane components, such as lipopolysaccharides (LPS) from *E. coli*, to incorporate chemical recognition into the imprinting process. This will allow the development of highly selective sensors capable of distinguishing not only bacterial species but, ideally, also strains with subtle surface differences.

Because the present SIP design primarily provides physical recognition, partial cross-reactivity may be expected for rod-shaped bacteria with dimensions close to *E. coli*; therefore, species-level discrimination among similarly shaped Gram-negative rods is not claimed at this stage. Accordingly, comprehensive mixed-species selectivity studies are most appropriately performed on the finalized receptor surface chemistry, where chemical cues (e.g., LPS-derived functionality) complement geometric matching and are expected to suppress cross-reactivity beyond morphology.

Ultimately, translation of SIP-based whole-cell sensors requires validation in complex matrices (e.g., milk, serum, food-process fluids), where possible refractive-index variability and non-specific adsorption can influence optical readouts and may necessitate matrix-aware protocols. Prior studies demonstrate that SIP/imprinted polymer-based sensors can remain functional in real matrices when modest sample handling is applied: e.g., *E. coli* detection in industrial rinse water and smoothie rinse water mixed with PBS (without filtration) with minimal preparation [38]. Moreover, molecularly imprinted polymer-based platforms have progressed toward *E. coli* sensing from real samples, including proof-of-concept measurements in milk, highlighting feasibility, as well as the importance of matrix-dependent optimization [71]. Importantly, our current assay already includes a post-incubation DI-water rinse prior to signal acquisition, which is expected to remove weakly bound species and salts and thereby reduce background contributions at the readout stage; together with the differential measurement design (measurement well versus PBS control well), this provides a practical basis for matrix-aware testing. To ensure that matrix effects are assessed on the sensor in its intended final configuration, comprehensive real-matrix validation is best performed once the receptor surface chemistry has been finalized (including the abovementioned LPS-based functionalisation to enhance *E. coli*-specific interactions and mitigate non-specific adsorption).

The reported findings in this study form a basis for future work dealing with a further miniaturization of the measurement setup (complete optical system) for being a point-of-care (POC) device, in particular. As a next step, by implementing individual light sources (e.g., light-emitting diodes (LEDs)) and a detector (such as a complementary metal-oxide-semiconductor (CMOS) camera), the intensity-based differential/multiplexed readouts with spatiotemporal resolution could be studied to eliminate the need for a spectrometer. In addition, ongoing research might deal with developing a smartphone-based POC device by employing the smartphone’s built-in LED flash as a light source and its CMOS camera as the detector [72,73].

## 4. Conclusions

This work reports on a surface-imprinted polymer (SIP)-based biomimetic sensor chip for *E. coli* detection. Using a “template bacteria-free” SIP sensor fabrication method, we reached 3 × 10^7^ biomimetic *E. coli* cavities per cm^2^. The developed SIP layer captured 2.1 times more *E. coli* bacteria than the NIP layer. More importantly, the adjusted distribution and orientation of *E. coli* cavities allows the SIP layer to serve as diffraction gratings that are used for optical detection of the bacteria. Studying the sensor’s *E. coli* detection event with FDTD simulations indicates that the sensing is related to a change in the refractive index upon binding the target bacteria within the cavities of the SIP surface. Finally, proof-of-concept experiments with differential signal measurements revealed the *E. coli* detection ability of the developed sensor chip, offering a new platform that is highly sensitive, low-cost, label-free, and quantitative. The developed two-in-one functioning concept could be suitable for the detection of other bacteria in future studies. Our group currently focuses on decorating the stamp surface with outer membrane components from the target bacteria (for example, lipopolysaccharides extracted from *E. coli* cells) to incorporate selective detection capabilities through “chemical recognition”. To facilitate the development of a field-deployable diagnostic platform enabling multiplexed and continuous bacteria measurements, future research should also concentrate on further miniaturization of the optical system and its advanced integration with complementary technologies such as smartphones, microfluidics, or CMOS cameras.

## Data Availability

The data presented in this study is available on request from the corresponding author.

## Supplementary Materials

The following supporting information can be downloaded at: https://www.mdpi.com/article/10.3390/bios16010060/s1, Section S1: Calculation details of differential measurements; Section S2: Details of the FDTD simulations; Figure S1: Videomicroscopic images of the sensor setup before and after analyte loading; Figure S2: SEM image comparing cell capture at the SIP/NIP border region; Figure S3: SEM images of *S. cerevisiae* and *P. megaterium* adhered to SIP surface; Figure S4: Relative intensity curves measured from SIP and NIP surfaces; Video S1: Visible reflected colors (rainbow) due to diffraction of broadband light on the receptor layer.

## References

[1] Alafeef M., Moitra P., Pan D. (2020). Nano-enabled sensing approaches for pathogenic bacterial detection. Biosens. Bioelectron..

[2] World Health Organization (2015). WHO Estimates of the Global Burden of Food-Borne Diseases.

[3] World Health Organization (2022). Food Safety.

[4] World Health Organization (2024). WHO Bacterial Priority Pathogens List, 2024.

[5] Abayasekara L.M., Perera J., Chandrasekharan V., Gnanam V.S., Udunuwara N.A., Liyanage D.S., Bulathsinhala N.E., Adikary S., Aluthmuhandiram J.V.S., Thanaseelan C.S. (2017). Detection of bacterial pathogens from clinical specimens using conventional microbial culture and 16S metagenomics: A comparative study. BMC Infect. Dis..

[6] Thakur B., Zhou G., Chang J., Pu H., Jin B., Sui X., Yuan X., Yang C.H., Magruder M., Chen J. (2018). Rapid detection of single *E. coli* bacteria using a graphene-based field-effect transistor device. Biosens. Bioelectron..

[7] Park K.S. (2018). Nucleic acid aptamer-based methods for diagnosis of infections. Biosens. Bioelectron..

[8] Shang Y., Xing G., Lin J., Li Y., Lin Y., Chen S., Lin J.M. (2024). Multiplex bacteria detection using one-pot CRISPR/Cas13a-based droplet microfluidics. Biosens. Bioelectron..

[9] Agar M., Laabei M., Leese H.S., Estrela P. (2025). Aptamer-molecularly imprinted polymer sensors for the detection of bacteria in water. Biosens. Bioelectron..

[10] Law J.W.F., Ab Mutalib N.S., Chan K.G., Lee L.H. (2015). Rapid methods for the detection of foodborne bacterial pathogens: Principles, applications, advantages and limitations. Front. Microbiol..

[11] Zhao Y., Zeng D., Yan C., Chen W., Ren J., Jiang Y., Jiang L., Xue F., Ji D., Tang F. (2020). Rapid and accurate detection of *Escherichia coli* O157:H7 in beef using microfluidic wax-printed paper-based ELISA. Analyst.

[12] Dinç S., Tjardts T., Maschkowitz G., Lukaszczuk V., Taghi Gharibzahedi S.M., Altintas Z. (2025). Highly sensitive detection of *Campylobacter jejuni* using a carbon dot-embedded nanoMIPs fluorescent sensor. Microchem. J..

[13] Zaraee N., Kanik F.E., Bhuiya A.M., Gong E.S., Geib M.T., Lortlar Ünlü N., Ozkumur A.Y., Dupuis J.R., Ünlü M.S. (2020). Highly sensitive and label-free digital detection of whole cell *E. coli* with interferometric reflectance imaging. Biosens. Bioelectron..

[14] Myndrul V., Arreguin-Campos R., Iatsunskyi I., Di Scala F., Eersels K., van Grinsven B. (2025). Photonic sensor based on surface imprinted polymers for enhanced point-of-care diagnosis of bacterial urinary tract infections. Biosens. Bioelectron..

[15] Uzun L., Turner A.P. (2016). Molecularly-imprinted polymer sensors: Realising their potential. Biosens. Bioelectron..

[16] Golabi M., Kuralay F., Jager E.W., Beni V., Turner A.P. (2017). Electrochemical bacterial detection using poly(3-aminophenylboronic acid)-based imprinted polymer. Biosens. Bioelectron..

[17] Yang Q., Li J., Wang X., Peng H., Xiong H., Chen L. (2018). Strategies of molecular imprinting-based fluorescence sensors for chemical and biological analysis. Biosens. Bioelectron..

[18] Cornelis P., Givanoudi S., Yongabi D., Iken H., Duwé S., Deschaume O., Robbens J., Dedecker P., Bartic C., Wübbenhorst M. (2019). Sensitive and specific detection of *E. coli* using biomimetic receptors in combination with a modified heat-transfer method. Biosens. Bioelectron..

[19] Yadav A.K., Verma D., Dalal N., Kumar A., Solanki P.R. (2022). Molecularly imprinted polymer-based nanodiagnostics for clinically pertinent bacteria and virus detection for future pandemics. Biosens. Bioelectron. X.

[20] Piletsky S., Canfarotta F., Poma A., Bossi A.M., Piletsky S. (2020). Molecularly imprinted polymers for cell recognition. Trends Biotechnol..

[21] Yilmaz E., Majidi D., Ozgur E., Denizli A. (2015). Whole cell imprinting based *Escherichia coli* sensors: A study for SPR and QCM. Sens. Actuators B Chem..

[22] Eersels K., Lieberzeit P., Wagner P. (2016). A review on synthetic receptors for bioparticle detection created by surface-imprinting techniques—from principles to applications. ACS Sens..

[23] Yongabi D., Khorshid M., Losada-Pérez P., Eersels K., Deschaume O., D’Haen J., Bartic C., Hooyberghs J., Thoelen R., Wübbenhorst M. (2018). Cell detection by surface imprinted polymers SIPs: A study to unravel the recognition mechanisms. Sens. Actuators B Chem..

[24] Iakimova T.M., Heidt B., Shen A.Q. (2024). Surface-imprinted polymers based on 3D printing resin for selective bacteria detection. Cell Rep. Phys. Sci..

[25] Chen L., Wang X., Lu W., Wu X., Li J. (2016). Molecular imprinting: Perspectives and applications. Chem. Soc. Rev..

[26] Caldara M., van Wissen G., Cleij T.J., Diliën H., van Grinsven B., Eersels K., Lowdon J.W. (2023). Deposition methods for the integration of molecularly imprinted polymers (MIPs) in sensor applications. Adv. Sens. Res..

[27] Werner M., Glück M.S., Bräuer B., Bismarck A., Lieberzeit P.A. (2022). Investigations on sub-structures within cavities of surface imprinted polymers using AFM and PF-QNM. Soft Matter.

[28] Özsoylu D., Aliazizi F., Wagner P., Schöning M.J. (2024). Template bacteria-free fabrication of surface imprinted polymer-based biosensor for *E. coli* detection using photolithographic mimics: Hacking bacterial adhesion. Biosens. Bioelectron..

[29] Latif U., Qian J., Can S., Dickert F. (2014). Biomimetic receptors for bioanalyte detection by quartz crystal microbalances—From molecules to cells. Sensors.

[30] Reshes G., Vanounou S., Fishov I., Feingold M. (2008). Cell shape dynamics in *Escherichia coli*. Biophys. J..

[31] Nečas D., Klapetek P. (2012). Gwyddion: An open-source software for SPM data analysis. Open Phys..

[32] Bertani G. (1951). Studies on lysogenesis. I. The mode of phage liberation by lysogenic *Escherichia coli*. J. Bacteriol..

[33] Schneider C.A., Rasband W.S., Eliceiri K.W. (2012). NIH Image to ImageJ: 25 years of image analysis. Nat. Methods.

[34] McCoy D.E., Shneidman A.V., Davis A.L., Aizenberg J. (2021). Finite-difference time-domain (FDTD) optical simulations: A primer for the life sciences and bio-inspired engineering. Micron.

[35] Liu P., Chin L., Ser W., Ayi T., Yap P., Bourouina T., Leprince-Wang Y. (2014). Real-time measurement of single bacterium’s refractive index using optofluidic immersion refractometry. Procedia Eng..

[36] Taya S.A., Alhamss D.N., Colak I., Patel S.K. (2022). Sensitivity enhancement of an optical sensor based on a binary photonic crystal for the detection of *Escherichia coli* by controlling the central wavelength and the angle of incidence. Opt. Quantum Electron..

[37] Long G.L., Winefordner J.D. (1983). Limit of detection a closer look at the IUPAC definition. Anal. Chem..

[38] Stilman W., Campolim Lenzi M., Wackers G., Deschaume O., Yongabi D., Mathijssen G., Bartic C., Gruber J., Wübbenhorst M., Heyndrickx M. (2022). Low cost, sensitive impedance detection of *E. coli* bacteria in food-matrix samples using surface-imprinted polymers as whole-cell receptors. Phys. Status Solidi A.

[39] Hui L., Chen J., Kafley P., Liu H. (2021). Capture and kill: Selective eradication of target bacteria by a flexible bacteria-imprinted chip. ACS Biomater. Sci. Eng..

[40] Li Q., Li R., Wang M., Wang J., Wang T., Jin Y., Li J., Wang F. (2025). Development of fluorescent artificial receptors for specific recognition and rapid detection of *Escherichia coli* O157:H7. Food Chem..

[41] van Grinsven B., Eersels K., Akkermans O., Ellermann S., Kordek A., Peeters M., Deschaume O., Bartic C., Diliën H., Steen Redeker E. (2016). Label-free detection of *Escherichia coli* based on thermal transport through surface imprinted polymers. ACS Sens..

[42] Arreguin-Campos R., Eersels K., Rogosic R., Cleij T.J., Diliën H., van Grinsven B. (2022). Imprinted polydimethylsiloxane-graphene oxide composite receptor for the biomimetic thermal sensing of *Escherichia coli*. ACS Sens..

[43] Choi I., Yang Y.I., Kim Y.J., Kim Y., Hahn J.S., Choi K., Yi J. (2008). Directed positioning of single cells in microwells fabricated by scanning probe lithography and wet etching methods. Langmuir.

[44] Hansen R.H., Timm A.C., Timm C.M., Bible A.N., Morrell-Falvey J.L., Pelletier D.A., Simpson M.L., Doktycz M.J., Retterer S.T. (2016). Stochastic assembly of bacteria in microwell arrays reveals the importance of confinement in community development. PLoS ONE.

[45] Zhu C., Luo X., Espulgar W.V., Koyama S., Kumanogoh A., Saito M., Takamatsu H., Tamiya E. (2020). Real-time monitoring and detection of single-cell level cytokine secretion using LSPR technology. Micromachines.

[46] Park M.C., Hur J.Y., Cho H.S., Park S.H., Suh K.Y. (2011). High-throughput single-cell quantification using simple microwell-based cell docking and programmable time-course live-cell imaging. Lab Chip.

[47] Cao J.T., Chen Z.X., Hao X.Y., Zhang P.H., Zhu J.J. (2012). Quantum dots-based immunofluorescent microfluidic chip for the analysis of glycan expression at single-cells. Anal. Chem..

[48] Kim S.H., Yamamoto T., Fourmy D., Fujii T. (2011). An electroactive microwell array for trapping and lysing single-bacterial cells. Biomicrofluidics.

[49] Park J., Park C., Sugitani Y., Fujii T., Kim S.H. (2022). An electroactive microwell array device to realize simultaneous trapping of single cancer cells and clusters. Lab Chip.

[50] Huang L., Liu Z., He J., Li J., Wang Z., Zhou J., Chen Y. (2023). AI-aided high-throughput profiling of single-cell migration and proliferation on addressable dual-nested microwell arrays. Cell Rep. Phys. Sci..

[51] Liu C., Liu J., Gao D., Ding M., Lin J.M. (2010). Fabrication of microwell arrays based on two-dimensional ordered polystyrene microspheres for high-throughput single-cell analysis. Anal. Chem..

[52] Jen C.P., Hsiao J.H., Maslov N.A. (2011). Single-cell chemical lysis on microfluidic chips with arrays of microwells. Sensors.

[53] Rettig J.R., Folch A. (2005). Large-scale single-cell trapping and imaging using microwell arrays. Anal. Chem..

[54] Helbig R., Günther D., Friedrichs J., Rößler F., Lasagni A., Werner C. (2016). The impact of structure dimensions on initial bacterial adhesion. Biomater. Sci..

[55] Umpleby R.J., Rushton G.T., Shah R.N., Rampey A.M., Bradshaw J.C., Berch J.K., Shimizu K.D. (2001). Recognition directed site-selective chemical modification of molecularly imprinted polymers. Macromolecules.

[56] Rushton G.T., Karns C.L., Shimizu K.D. (2005). A critical examination of the use of the Freundlich isotherm in characterizing molecularly imprinted polymers (MIPs). Anal. Chim. Acta.

[57] Regan B., Boyle F., O’Kennedy R., Collins D. (2019). Evaluation of molecularly imprinted polymers for point-of-care testing for cardiovascular disease. Sensors.

[58] Muslihati A., Wulandari C., Septiani N.L.W., Gumilar G., Subagio A., Hamidah I., Nugraha N., Peiner E., Wasisto H.S., Yuliarto B. (2025). High-performance electrochemical biosensor comprising Mn-ZIF-67 conjugated with anti-O antibody for *Escherichia coli* detection. Commun. Chem..

[59] Wang M., Zheng L., Sun F., Ye Q., Liang P., Pang K., Ye Z., Wang Y. (2025). Revolutionizing *Escherichia coli* detection in real samples with digital SERS aptamer sensor technology. Spectrochim. Acta Part A Mol. Biomol. Spectrosc..

[60] Tripathi S.M., Bock W.J., Mikulic P., Chinnappan R., Ng A., Tolba M., Zourob M. (2012). Long period grating based biosensor for the detection of *Escherichia coli* bacteria. Biosens. Bioelectron..

[61] Barrios C., Zhenhe C., Navarro-Villoslada F., López-Romero D., Moreno-Bondi M. (2011). Molecularly imprinted polymer diffraction grating as label-free optical bio(mimetic)sensor. Biosens. Bioelectron..

[62] Elsherif M., Hassan M.U., Yetisen A.K., Butt H. (2018). Wearable contact lens biosensors for continuous glucose monitoring using smartphones. ACS Nano.

[63] Pereira C.F., Sales M.G.F., Frasco M.F. (2022). A molecularly imprinted photonic polymer based on an inverse opal structure for sensing D-dimer at the point-of-care. Talanta.

[64] Wen X., Liu Y., Liu Q., Chen Z., Hu X., Xu C., Chen H., Xing M., Qu H., Zhang M. (2022). Glucose sensing based on hydrogel grating incorporating phenylboronic acid groups. Opt. Express.

[65] Resende S., Fernandes J., Sousa P.C., Calaza C., Frasco M.F., Freitas P.P., Goreti F., Sales M. (2024). Fabrication and sensing properties of a molecularly imprinted polymer on a photonic PDMS substrate for the optical detection of C-reactive protein. Chem. Eng. J..

[66] Hayden O., Lieberzeit P.A., Blaas D., Dickert F.L. (2006). Artificial antibodies for bioanalyte detection—sensing viruses and proteins. Adv. Funct. Mater..

[67] Hayden O., Mann K., Krassnig S., Dickert F.L. (2006). Biomimetic ABO blood-group typing. Angew. Chem. Int. Ed..

[68] Murugan D., Tintelott M., Narayanan M.S., Vu X., Kurkina T., Rodriguez-Emmenegger C., Schwaneberg U., Dostalek J., Ingebrandt S., Pachauri V. (2024). Recent advances in grating coupled surface plasmon resonance technology. Adv. Opt. Mater..

[69] Birhanu Hayilesilassie R., Gemta A.B., Maremi F.T., Getahun Kumela A., Gudishe K., Dana B.D. (2024). Detection and photothermal inactivation of gram-positive and gram-negative bloodstream bacteria using photonic crystal biosensor and plasmonic core–shell. RSC Adv..

[70] Pepino R., Tari H., Bile A., Nabizada A., Fazio E. (2025). Optical bacteria recognition: Cross-polarized scattering. Symmetry.

[71] Arreguin-Campos R., Eersels K., Lowdon J.W., Rogosic R., Heidt B., Caldara M., Jiménez-Monroy K.L., Diliën H., Cleij T.J., van Grinsven B. (2021). Biomimetic sensing of *Escherichia coli* at the solid-liquid interface: From surface-imprinted polymer synthesis toward real sample sensing in food safety. Microchem. J..

[72] Wang Y., Liu X., Chen P., Tran N.T., Zhang J., Chia W.S., Boujday S., Liedberg B. (2016). Smartphone spectrometer for colorimetric biosensing. Analyst.

[73] Wang L.J., Chang Y.C., Ge X., Osmanson A.T., Du D., Lin Y., Li L. (2016). Smartphone optosensing platform using a DVD grating to detect neurotoxins. ACS Sens..

