# Supplementary material for: Surface-Imprinted Polymer Coupled with Diffraction Gratings for Low-Cost, Label-Free and Differential E. coli Detection
Source: Biosensors (Basel). 2026 Jan 13;16(1):60. doi: 10.3390/bios16010060 (PMC12839240; doi:10.3390/bios16010060)
Supplement: Supplementary file 1 [file biosensors-16-00060-s001.zip › Supplementary Information.pdf]

Supplementary Information

Surface-Imprinted Polymer Coupled with Diffraction Gratings for Low-Cost, Label-Free and Differential *E. coli* Detection

*Dua Özsoylu [a], Elke Börmann-El-Kholy [a], Rabia Nur Kaya [a], Patrick Wagner [b], Michael J. Schöning [a, c, *]*

[a] *Institute of Nano- and Biotechnologies (INB), Aachen University of Applied Sciences, Campus Jülich, 52428, Jülich, Germany*

[b] *Laboratory for Soft Matter and Biophysics, Department of Physics and Astronomy, KU Leuven, Celestijnenlaan 200 D, B-3001 Leuven, Belgium*

[c] *Institute of Biological Information Processing (IBI-3), Research Centre Jülich GmbH, Wilhelm-Johnen-Straße, 52428 Jülich, Germany*

* Corresponding author: Prof. Dr. Michael J. Schöning
Email: schoening@fh-aachen.de

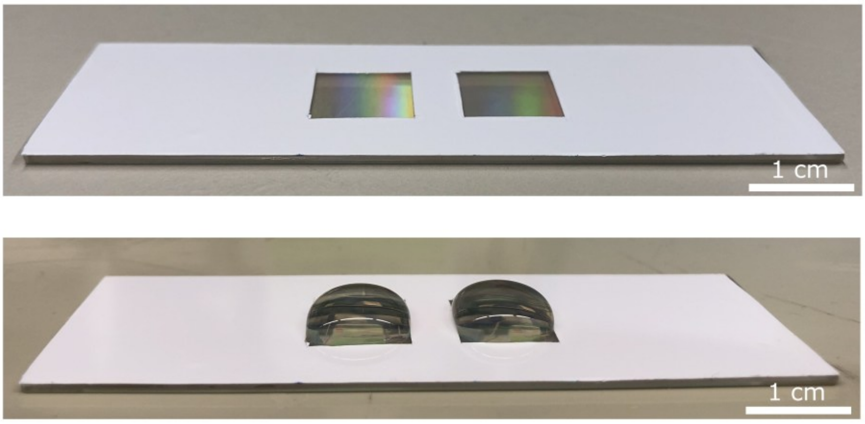

Fig. S1: Videomicroscopic images of the sensor setup before (top) and after (bottom) loading with analyte solution (300 µL) and showing the ability to hold the solution thanks to the hydrophobic barrier provided by the tape (white layer).

S1. Calculation details of differential measurements

For differential measurements, each curve related to the *E. coli* measurement (see, Figure 6a in the main text) was subtracted from the curve of "reference measurement" (initial signal without any incubation on the wells). This way, the "relative intensity change" *versus* "wavelength" curves were obtained.

As a result, to quantify the spectral changes induced by *E. coli* and to compensate for possible sensor drift, the drift-compensated relative intensity change, $\Delta I_{\text{corrected}}(\lambda)$ was calculated as (see Eq. (S1)):

$$\Delta \mathrm{I}_{\text{corrected}}(\lambda) = \left[\mathrm{I}_{E.coli}(\lambda) - \mathrm{I}_{\text{ref},E.coli}(\lambda)\right] - \left[\mathrm{I}_{\text{control}}(\lambda) - \mathrm{I}_{\text{ref,control}}(\lambda)\right] \quad \text{(S1)}$$

where:

λ is the wavelength,
$I_{E.coli}(\lambda)$ is the measured spectral intensity from the *E. coli* well after incubation,
$I_{\text{ref},E.coli}(\lambda)$ is the corresponding reference spectrum from the same well before incubation,
$I_{\text{control}}(\lambda)$ is the spectral intensity from the control well (PBS only) after incubation, and
$I_{\text{ref,control}}(\lambda)$ is the reference spectrum of the control well before incubation.

Hence, the "relative intensity change" *versus* "wavelength" curves can be obtained. A clear drop at relative intensity in the curves can be observed at the wavelength range of 660-700 nm over *E. coli* cell admission. Therefore, calibration curves (relative intensity change *versus* applied cell concentration) were obtained by linear fitting the intensity changes corresponding to this wavelength range (from 660 nm to 700 nm, a total of 261 points). All measurements and incubation steps were performed at room temperature inside a dark Faraday cage.

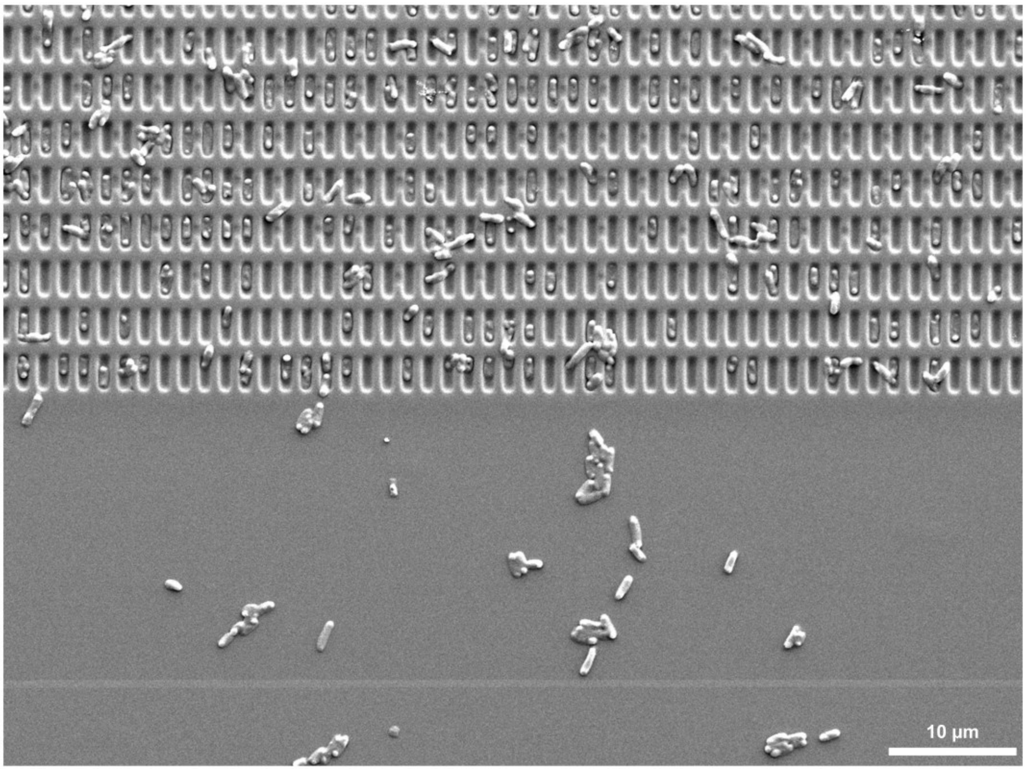

Fig. S2: SEM image showing the variation in cell capturing between SIP- (upper, patterned area) and NIP-based (lower, non-patterned flat area) surfaces at the border regions (where imprinted and non-imprinted surfaces meet).

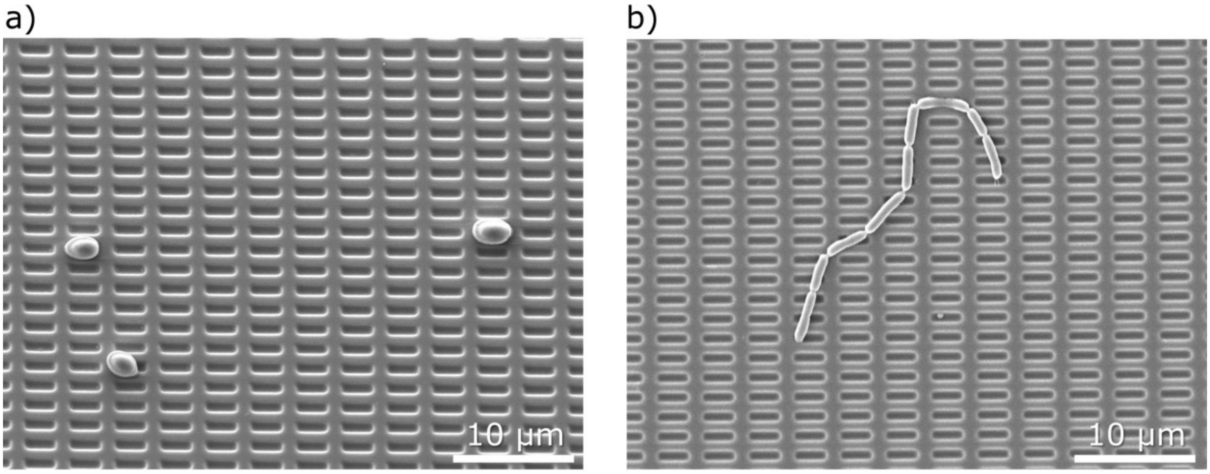

Fig. S3: SEM images showing a) *S. cerevisiae* and b) *P. megaterium* cells adhered to a SIP surface, which is adapted to the geometry of *E. coli* cells, after 15 min exposure of 5×10^8 cells/mL of *S. cerevisiae* and *P. megaterium*, respectively, in PBS.

S2. Details of the FDTD simulations

S2.1 Reason for selecting Bloch boundary condition (instead of the periodic boundary condition)

As recommended by the manufacturer of the simulatioun software (Lumerical, Ansys Inc., USA), we used Bloch boundary condition instead of periodic boundary condition. In periodic boundary condition, the fields at one edge of the simulation region are simply copied and then, they are re-injected at the other edge. However, when the propagation is at an angle (45° in our simulation set-up), the fields from one period to the next are not precisely periodic. This causes the fields going out of phase by some amount. Bloch boundary condition (BC) works similarly; however, it also applies a phase correction to the fields that are very similar, while it copies the fields from one edge to the other. Therefore, we utilized Bloch boundary condition instead of periodic boundary condition in the X and Y directions.

S2.2 Details of simulation work for reflectance changes as a function of wavelength

Due to the organization of the reflection probe in the real measurement set-up, in which both spectrometer fiber and illumination fibers are bundled in the same location, only the light reflected back with the same angle of its incident angle will be measured. Also here, the reflectance change (over *E. coli* admission) at an incident angle (45° of 4th diffraction order in the case above) is considered. For this, a 2D simulation with the best mesh refinement option (mesh accuracy parameter of factor 8 that is the highest number allowed by the Lumerical Software) at the cross-sectional XZ plane towards the middle of the cavity was performed. This configuration decreases the memory requirement, resulting in faster simulation outcomes, while providing higher simulation accuracy. To find out the reflectance of the specific order where the reflected light goes back with the same angle of its injection angle, the following grating script commands of the Lumerical software were applied:

```
run;
mname="Reflection";            # monitor name
theta=gratingangle(mname);   # angle of each grating order
G=grating(mname);              # power to each order (fraction of transmitted power)

plot(theta,G,"theta (deg)","relative power","grating orders","plot points");
?G;                            # calculate relative power of each order

mname="Reflection";            # monitor name
f=getdata(mname,"f");          # get frequency vector
T=transmission(mname);       # get total reflection
?T;                            # calculate total reflection
?T*G;                          # calculate reflected power of each order
```

Considering the narrow wavelength range (around 535-700 nm) of the spectrometer and 45° of the incident light, only the source injected with around 584 nm can be received by the spectrometer fiber as part of the 4th diffraction order (as shown above), while others have different (but sequential) angles to give a spectrum in the same order. Thus, under normal circumstances, it is expected to have a peak at 584 nm on the intensity spectra. However, to simplify the setup for potential future applications (such as smartphone-based biosensor design), both an optical collimator lens and a polarizer were not implemented in our

experimental set-up. Therefore, incident light with broader angles also interacts with the SIP surface. Hence, we first calculated each wavelength for each possible incident angle (from 31° to 58°) where the reflected light goes back with the same angle. Thereafter, a separate simulation was performed for this particular wavelength value to extract the reflectance. In addition, separate simulations were carried out with both polarization angles of 0° (p-polarized radiation) and 90° (s-polarized radiation). Reflectance values from the simulations of p- and s-polarization were averaged to obtain the reflection of unpolarized light. When the wavelength ranges from different angles of incident light are overlapped, the sum of their reflectance was calculated.

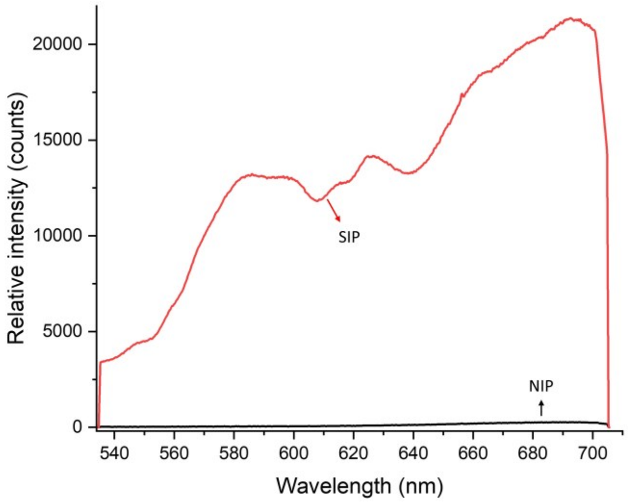

Fig. S4: Relative intensity curves measured from SIP and NIP surface, respectively.
